# The Making of a Trained Nurse: Possible Changes Due to the War

**Published:** 1918-05-04

**Authors:** 


### THE MAKING OF A TRAINED NURSE.

### Possible Changes Due to the War.

It is no part or purpose of the present article to inquire closely into the possible reasons, if any, other than the war which may underlie the shortage in probationers at Whitechapel. Many hospitals are threatened with the same difficulties, and, naturally, the high repute or otherwise of an institution must necessarily tend to diminish or increase the trouble. Be the cause what it may, the authorities at the London Hospital have determined to make a new departure, which is sure to be criticised, and the results of which will be watched with no small interest in many quarters. In the third year of the war, and since, the self-denying and efficient service rendered in their work in connection with hospitals by V.A.D.s has become universally admitted and appreciated. So much is this the case that sisters and trained nurses of the highest type have borne spontaneous and independent testimony to the valuable services rendered by the best of the V.A.D.s, and to the claim they have established for generous recognition on the part of all who are responsible for the adequate staffing and efficient nursing of our hospitals at the seat of war, and in many of the hospitals in the Homeland also. In these circumstances the London Hospital authorities have determined to take as probationers for training those V.A.D.s whose hospital work has shown their aptitude for this vocation, and who have acquitted themselves with declared efficiency in the fields of work opened to them and in the discharge of the duties which have devolved upon them as assistants in various capacities in Military Territorial, and other hospitals at home and abroad. The London, therefore, offers to take for training a number of V.A.D.s who, for one or two previous years, have so proved themselves efficient, and to admit them as probationers, providing they agree to undergo a course for two further years of complete training as probationer nurses at the London. In the case of V.A.D.s who pass the requisite examinations, which will be identical with those at present held for the testing of ordinary probationer nurses, it is agreed to give them, when successful in their examinations, a three years' certificate of training at the end of the two years' course. We have not space in the present article to go fully into the details of the plan, but the outline here given will sufficiently indicate the basis of the arrangement, which, without altering the course of training at present in force at the London, will enable V.A.D.s of the highest type to qualify themselves as trained nurses after two years' training, plus their time as V.A.D.s.

In this connection it is interesting to note that the *Canadian Nurse* for March and the *American Journal of Nursing* for April each contains an article by Isabel M. Stewart, R.N., on possible changes in the department of nursing education to meet the war emergency. The *American Journal* explains: "The laws to be amended provide *in California* that an accredited training-school for nurses within the meaning of this Act (Registration of Nurses) is defined to be a school for the training of nurses in connection with a hospital or hospitals giving a general training and a systematic theoretical and practical course of instruction covering a period of at least three years. *In Maryland* the law provides that each applicant shall furnish evidence to the Board of Examiners that she has graduated from a training-school connected with a general hospital where three years of training and a systematic course of instruction is given in the hospital." Miss Stewart explains, "it is not suggested to break down a three years' law and substitute a two years' law for it," but "to offer such an amendment to the law, as would allow greater flexibility of application and more liberal interpretation of its requirements." Instead of three full years of training in a hospital, it is proposed to allow some portion of this time to be spent in preparation in an approved college or technical school, or in special training for public health nursing or social service, or in service in military hospitals in time of war. It is maintained that this freer interpretation of the three-year law "would not be breaking down standards of nursing education," but "would rather be providing for certain developments in the present American system which would not be possible otherwise." It is interesting to compare the changes to be made at the London Hospital with the proposals in the United States to meet the war emergency which is threatened by the anticipated shortage of nurses for war service and training.

